# Explainable deep learning model for automatic mulberry leaf disease classification

**DOI:** 10.3389/fpls.2023.1175515

**Published:** 2023-09-19

**Authors:** Md. Nahiduzzaman, Muhammad E. H. Chowdhury, Abdus Salam, Emama Nahid, Faruque Ahmed, Nasser Al-Emadi, Mohamed Arselene Ayari, Amith Khandakar, Julfikar Haider

**Affiliations:** ^1^ Department of Electrical & Computer Engineering, Rajshahi University of Engineering & Technology, Rajshahi, Bangladesh; ^2^ Department of Electrical Engineering, Qatar University, Doha, Qatar; ^3^ Bangladesh Sericulture Research and Training Institute, Rajshahi, Bangladesh; ^4^ Department of Civil and Environmental Engineering, Qatar University, Doha, Qatar; ^5^ Technology Innovation and Engineering Education Unit, Qatar University, Doha, Qatar; ^6^ Department of Engineering, Manchester Metropolitan University, Manchester, United Kingdom

**Keywords:** mulberry leaf, depth wise separable convolution, parallel convolution, explainable artificial intelligence (XAI), Shapley Additive Explanations (SHAP)

## Abstract

Mulberry leaves feed Bombyx mori silkworms to generate silk thread. Diseases that affect mulberry leaves have reduced crop and silk yields in sericulture, which produces 90% of the world’s raw silk. Manual leaf disease identification is tedious and error-prone. Computer vision can categorize leaf diseases early and overcome the challenges of manual identification. No mulberry leaf deep learning (DL) models have been reported. Therefore, in this study, two types of leaf diseases: leaf rust and leaf spot, with disease-free leaves, were collected from two regions of Bangladesh. Sericulture experts annotated the leaf images. The images were pre-processed, and 6,000 synthetic images were generated using typical image augmentation methods from the original 764 training images. Additional 218 and 109 images were employed for testing and validation respectively. In addition, a unique lightweight parallel depth-wise separable CNN model, PDS-CNN was developed by applying depth-wise separable convolutional layers to reduce parameters, layers, and size while boosting classification performance. Finally, the explainable capability of PDS-CNN is obtained through the use of SHapley Additive exPlanations (SHAP) evaluated by a sericulture specialist. The proposed PDS-CNN outperforms well-known deep transfer learning models, achieving an optimistic accuracy of 95.05 ± 2.86% for three-class classifications and 96.06 ± 3.01% for binary classifications with only 0.53 million parameters, 8 layers, and a size of 6.3 megabytes. Furthermore, when compared with other well-known transfer models, the proposed model identified mulberry leaf diseases with higher accuracy, fewer factors, fewer layers, and lower overall size. The visually expressive SHAP explanation images validate the models’ findings aligning with the predictions made the sericulture specialist. Based on these findings, it is possible to conclude that the explainable AI (XAI)-based PDS-CNN can provide sericulture specialists with an effective tool for accurately categorizing mulberry leaves.

## 1 Introduction

Agricultural production, like any other industry, assists farmers in securing their financial future. Agricultural expansion is critical for a powerful nation because it meets a real need in the global economy and ensures sustainability. Plants are susceptible to disease at various phases of development, just like humans. As a result, the farmer’s overall crop output and revenue suffer. With the global population expected to exceed 9 billion by 2050, developing innovative methods for identifying and mitigating plant diseases can increase food supplies while reducing the demand for pesticides (Sharma et al., 2020). Early detection and analysis of the several forms of illnesses that might harm a crop are critical to the profitability of the agriculture business. Traditionally, conventional methods for diagnosing and identifying plant diseases have relied on professional observation with only their naked eyes. Furthermore, this method can be tedious, long, and expensive, rendering it unsustainable for millions of small and medium-sized farms around the world. As a result, there is a significant danger of the illness spreading to other healthy plants. To address these challenges, researchers from around the world have presented cutting-edge automated systems that use machine learning (ML) and deep learning (DL) techniques to detect diseases in various plants such as rice/paddy (Lu et al., 2017; Malvade et al., 2022), tomato (Hassanien et al., 2017; Ireri et al., 2019), cotton (Ferentinos, 2018; Patil and Patil, 2021), watermelon (Pantazi et al., 2019; Singh, 2019), and sunflower (Singh, 2019) from various parts of the plants, particularly their leaves (Singh and Misra, 2017; Dhingra et al., 2019; Pantazi et al., 2019). The bulk of the experiments described used leaf photos from the Plant Village dataset as well as real-time data comparable to these plants. Furthermore, the authors (Ferentinos, 2018) classified plant illnesses using 58 different plant species.

**Figure 1:**
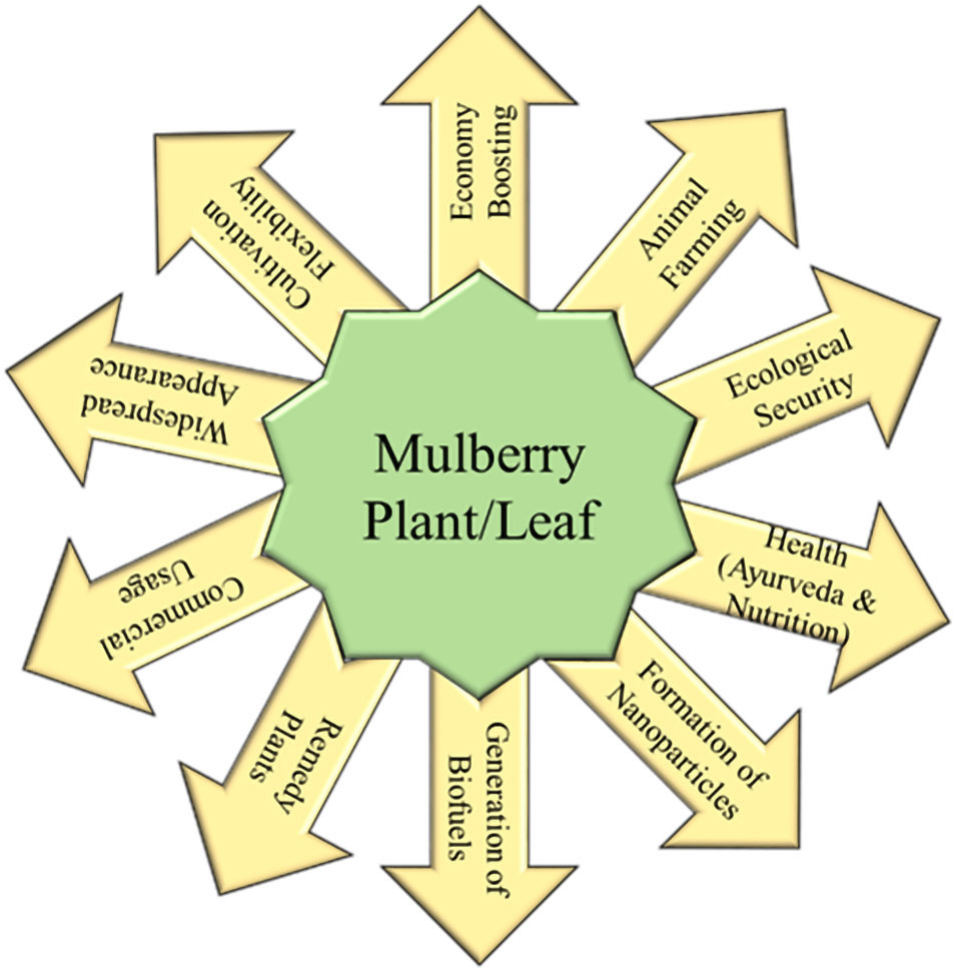
The mulberry's varied applications in many scenarios.

The mulberry (Morus spp.), a member of the Moraceae family, is a fast-growing, deciduous woody tree species native to India and China’s Himalayan foothills (Yuan and Zhao, 2017). Mulberry is economically valuable because silkworm larvae (Bombyx mori) feed on its leaves to make mori silk. Mulberry leaves are also fed to animals. Despite its long history of use in silk manufacture and animal husbandry, the ecological relevance of mulberry has been underestimated. In recent years, there has been a broad acknowledgement of this plant’s effectiveness in a variety of disciplines, including environmental safety, medicine, and industry (Rohela et al., 2020). **Figure 1** depicts the various applications of mulberry. Mulberry leaves, on the other hand, are widely utilized in the raising of the silkworm, whose cocoons are spun into silk yarn. The silkworm produces silk protein by using the protein found in mulberry leaves (namely, fibroin and sericin). The silk fiber produced is used in the commercial production of high-quality silk apparel. Mulberry trees provide up to 90% of the world’s raw silk supply, and their cultivation is critical to the economic well-being of countless people, particularly in India and Bangladesh (Chowdhury et al., 2017). Because of the mulberry leaf’s economic importance in sericulture, the quality and amount of leaf produced per unit area have a direct impact on silk cocoon yield.

Sericulture has been identified as a viable new economic driver in Bangladesh. Bangladesh has a huge possibility to develop major economic growth in this sector with the assistance of the government and non-governmental organizations. To further the study of sericulture, the government of Bangladesh founded the Bangladesh Sericulture Research and Training Centre in Rajshahi. The Bangladesh Sericulture Board was founded in 1977 by Ordinance No. 62, signed by the President of the People’s Republic of Bangladesh (Chowdhury et al., 2017). Only two sub-districts (Bholahat and Shibganj) in the current Nawabganj district of the Rajshahi division were initially suited for sericulture. Since Bangladesh’s independence, sericulture has extended to 36 districts out of a total of 64 (Banglapedia, 2021), contributing to poverty reduction and increased employment prospects throughout the country, particularly in rural areas.

Yet, sericulture can only be successful if the silkworms are fed fresh mulberry leaves. Mulberry plants are vulnerable to several fungal diseases, the most frequent of which are Cercospora moricola’s leaf rust and leaf spot infections (Banglapedia, 2021). Mulberry plants are vulnerable to a variety of pests, including the hairy caterpillar (Spilarctia obliqua), among others. Diseases and pests typically cause a considerable decrease in mulberry leaf yield, which leads to a decrease in silk production. As a result, farmers are incurring enormous economic losses, which has a detrimental influence on Bangladesh’s national economy (Rashid et al., 2014). Furthermore, silk gowns have traditional importance in specific areas of Bangladesh; thus, the loss in silk manufacturing has been exacerbated by the use of various synthetic fibers. If this problem is not addressed properly, future generations will be unaware of this traditional product and its aesthetic worth, and those affiliated with this sector will be unemployed.

However, no study has been conducted to the best of the authors’ knowledge to identify mulberry illnesses from their leaves using image classification. Similarly, because there was no existing image dataset for mulberry leaves, photos of mulberry leaves were obtained in Rajshahi, Bangladesh, for this research to establish a unique dataset, which was manually categorized by a sericulture specialist into “healthy,” “leaf spot,” and “leaf rust.” Only two diseases were considered for this research because they are common in Bangladesh during the winter season, and the photos were obtained during the winter months in Bangladesh. The images are then preprocessed using various methodologies, and a novel lightweight parallel PDS-CNN model for disease classification in mulberry leaves is constructed based on those processed images. It’s also worth noting that, to the best of the authors’ knowledge, no studies have assessed the interpretability capabilities of ML/DL models using SHapley Additive exPlanations (SHAP) or local interpretable model-agnostic explanations (LIME) in this area of the agricultural industry (classify distinct plant ailments from leaf images). As a result, this study provided a novel framework based on explainable artificial intelligence (XAI) that demonstrated which sections of a picture the proposed model paid significantly more attention to than the others. The unique, lightweight model provided here can be put into an embedded system to assist farmers in the field in the early diagnosis of mulberry diseases, avoiding crop loss and ensuring the production of healthy leaves for several applications. The major contributions of this work are as follows:

1. For the first time, a unique dataset of 1,091 annotated mulberry leaf images (Healthy: 440, Leaf Rust: 489, and Leaf Spot: 162) was created as no such datasets are available in the existing open sources.
2. To assure the building of a strong AI model with no overfitting concerns, synthetic data was generated using standard data augmentation methods.
3. To classify mulberry diseases, a novel lightweight parallel depthwise separable convolutional neural network (PDS-CNN) model was developed through customization of existing lightweight CNN model.
4. The classification performance, as well as the parameters, layers, and sizes of the models, were compared to various well-known transfer learning (TL) models.
5. For the first time, the interpretability capacity of the proposed framework has been exhibited using SHAP to ensure the model is correctly focused on the area of interest.

## 2 Literature review

Scientists have recently devised unique approaches for the automatic identification and classification of numerous plant diseases. This section investigates and reviews some of these strategies. To detect bacterial spot disease in peach leaf images, Bedi et al. created a hybrid model by combining a convolutional auto-encoder and a convolutional neural network (CNN) (Bedi and Gole, 2021). The PlantVillage dataset provided the researchers with 4,457 leaf images (healthy: 2,160 and bacterial spot: 2,297), with 70% of the images utilized for training and 30% used to calculate the model’s performance, which reached an accuracy of 98.38%. Similarly, Akbar et al. suggested a VGG-19-based lightweight CNN (LWNet) model identify and categorize peace leaf images into healthy and bacteriosis images (Akbar et al., 2022). Initially, they photographed 625 healthy and 375 diseased leaf images from a research farm at Pakistan’s Agricultural University in Peshawar. Subsequently, using these genuine leaf images, 10,000 (Healthy: 5,500 and bacterial spot: 4,500) synthetic data points were generated, which were then subjected to image pre-processing procedures such as image scaling, noise removal, and background removal. Eventually, 7,000 images were used to train their model, and 3,000 images were utilized to test the LWNet model, resulting in an accuracy of 98.87% and a simulation time of 1 hour 56 minutes and 38 seconds.

Lu et al. developed a deep CNN model to recognize ten types of rice diseases from images of rice plant leaves (Lu et al., 2017). They obtained 500 images from the Heilongjiang Academy of Land Reclamation Sciences in China with the Canon EOS 5D Mark III, then selected 10,000 12×12 patches from these photographs to train their CNN model. They also ran 10-fold cross-validation (CV) on their model and compared the results for different filter sizes, attaining the greatest accuracy of 95.48%. Additionally, Ramesh et al. suggested a deep neural network optimized with the Jaya algorithm (DNN JOA) to classify four types of rice plant diseases from rice plant leaves (Ramesh and Vydeki, 2020). The 650 leaf images (healthy: 95, bacterial blight: 125, blast: 170, sheath rot: 110, and brown spot: 150) were taken in the rural areas of Ayikudi and Panpoli, Tirunelveli District, Tamilnadu, using a high-resolution digital camera (DC). The authors used a clustering algorithm to separate the diseased, normal, and background sections. The DNN JOA model was trained using 70% of the images, 10% of the images were used for validation, and 20% of the images were used to test the proposed model. The model achieved an overall accuracy of 97% after 125 epochs. Anami et al. employed a pre-trained transfer learning (TL) model, VGG-16, to automatically classify 12 forms of stress from paddy plant leaf images (Anami et al., 2020). The proposed VGG-16 model was trained using 3,600 images and tested using 2,400 images, with average stress classification accuracies of 90.75%, 93.38%, 93.135, 95.08%, 92.13%, and 92.89% for Jaya, Abhilasham, Mugad Suganda, Mugad 101, and Mugad Siri, respectively. Malvades et al. also collected 3,355 paddy leaf photos (healthy: 1,488, brown spot: 523, hispa pests: 565, leaf blast: 779) and then used several common image augmentation techniques to reduce overfitting (Malvade et al., 2022). They compared five TL models for the classification of paddy crop stresses; among them, the ResNet-50 model showed promising accuracy of 92.61% while training 70% of all images in 1,626 seconds.

Gonzalez-Huitron et al. used four TL models: MobileNetV2, NasNetMobile, Xception, and MobileNetV3 to diagnose 10 different tomato leaf diseases from images of tomato leaves (Gonzalez-Huitron et al., 2021). A total of 109,290 images were obtained from 18,215 images in the PlantVillage dataset utilizing data augmentation, and 30% of the data was used to test their models, with the best accuracy of nearly 100% attained using Xception at 2,512 seconds per epoch. Furthermore, they deployed their model on the Raspberry Pi 4 single-board computer. Abbas et al. used the PlantVillage dataset with a TL model called DenseNet121 to detect several types of tomato leaf diseases (Abbas et al., 2021). To begin, the authors used a conditional generative adversarial network (771,454 trainable parameters) to create 4,000 synthetic tomato leaf images, which were then mixed with 16,012 genuine pictures. The DenseNet121 was then fine-tuned by replacing the top fully connected (FC) layer and softmax layer with convolutional layers (CL) with the ReLU activation function, followed by an FC layer and a softmax layer to classify ten different forms of tomato leaf diseases. For the 5, 7, and 10 classes, the model achieved 99.51%, 98.65%, and 97.11%, respectively. Similarly, Chowdhury et al. used 18,161 tomato leaf pictures from the PlantVillage dataset to detect tomato leaf illnesses using three TL models: EfficientNet-B0, B4, and B7 (Chowdhury et al., 2021). They used various augmentation approaches to balance the data and reduce overfitting because the dataset was not balanced. They tested their models on 20% of tomato leaf images, and EfficientNet-B4 achieved the maximum accuracy with 99.95%, 99.12%, and 99.89% for 2, 6, and 10-class classification, respectively.

To detect three types of grape diseases from leaf images, Ji et al. developed a combined CNN model based on two TL models: GoogleNet and ResNet50 (Ji et al., 2020). The PlantVillage collection yielded a total of 1,619 grape leaf images (healthy: 171, black rot: 476, esca: 552, and isariopsis leaf spot: 420). The authors used GoogLeNet and ResNet to extract features from leaf images, and the features from these two models were concatenated before being sent into the Fully connected layers and a SoftMax layer to discriminate these diseases from healthy leaf images. The suggested UnitedModel achieved 98.57% accuracy, 99.05% precision, and 98.88% recall, on average. Paymode et al (Paymode and Malode, 2022). used a VGG16 model to detect numerous crop leaf diseases from images of tomato and grape leaves. Farmers from Aurangabad, India, collected a total of 14,421 tomato leaf images (early blight: 1,000, mosaic virus: 373, bacterial spot: 2,127, late blight: 1,909, leaf mould: 952, septoria leaf spot: 1,771, spot: 1,404, spider mites: 1,676, and yellow leaf curf: 3,207). Following that, they used various image processing methods such as filtering, grayscale transformation, data augmentation, and so on. Lastly, for grape and tomato leaf disease classification, the VGG16 model was trained for 40 and 30 epochs, respectively, and attained an accuracy of 98.40% and 95.71%.

Ferentinos used several TL models including as AlexNet, GoogLeNet, VGG, etc to detect 58 specific diseases of various plants such as oranges, apples, onions, watermelons, strawberries, and soybeans from leaf images (Ferentinos, 2018). A total of 87,848 leaf images of healthy and diseased plants were collected, with 70,300 utilized to train their models and 17,548 used to test the suggested models. The VGG model achieved an optimistic accuracy of 99.48% after 48 epochs at a time of 7,294 seconds per epoch. Furthermore, the model classifies every single image in 2 milliseconds on a single graphics processing unit (GPU). Zhang et al., on the other hand, used a BM-500GE digital camera to capture 700 images of cucumber leaves (healthy: 100, downy mildew: 100, anthracnose: 100, grey mold: 100, angular leaf spot: 100, black spot: 100, and powdery mildew: 100). The author presented a global pooling dilated CNN for the classification of six types of cucumber leaf diseases and achieved an accuracy of 94.65%, with training and testing times of 6.2 hours and 3.58 seconds, respectively. Singh classified seven forms of sunflower leaf diseases from leaf images using a particle swarm optimization (PSO) technique (Singh, 2019). Median filtering improved the quality of leaf images, and the PSO algorithm reached an accuracy of 98%. Ayalew et al. used Gabor wavelet characteristics to classify scenes in wild blueberry fields (Ayalew et al., 2021). The authors used an IDSEye 1220SE/C industrial camera to acquire 3,281 images from five fields, and the classification accuracy for each field was between 87.9% and 98.3%, with a total of 27 to 72 Gabor features used. Raouhi et al. classified seven olive disorders, including healthy images, using seven TL models such as EffiecientNetB7, InceptionV3, VGG19, ResNet50, and others with four activation functions: Adam, Adagrad, SGD, and Rmsprop (Raouhi et al., 2022). The scientists collected 5,571 images of olive leaves from various parts of Morocco and employed several data augmentation approaches to deal with the overfitting problem. Using MobileNet with the Rmsprop function, 20% of the data used to test their various models attained the best accuracy of 98.43%. **Table 1** outlines various leaf disease classification methods and associated performance factors.

**Table 1:** Summary of the state-of-art models.

References	Dataset	Best Model	Testing Accuracy	Best Model’s Parameters (Million)	XAI
(Mohanty et al., 2016)	PlantVillage (14 Plants’ Leaves)	GoogLeNet	99.3%	7	No
(Ferentinos, 2018)	Custom (58 Plants’ Leaves)	VGGNet	99.48%	138.4	No
(Zhang et al., 2019)	Custom (Cucumber Leaves)	Custom CNN	94.65%	–	No
(Chohan et al., 2020)	PlantVillage (14 Plants’ Leaves)	VGG-19	98.3%	143	No
(Sanga et al., 2020)	Custom (Banana Leaves)	ResNet-152	99.2%	60	No
(Abbas et al., 2021)	PlantVillage (Tomato Leaves)	DenseNet121	97.11%	8.1	No
(Chowdhury et al., 2021)	PlantVillage (Tomato Leaves)	EfficientNet-B4	99.95%	19.5	No
(Paymode and Malode, 2022)	Custom (Grape and Tomato Leaves)	VGG16	Grape: 98.40% and Tomato: 95.71%,	138.4	No
(Akbar et al., 2022)	Custom (Peace Leaves)	LWNet	98.87%	–	No
(Raouhi et al., 2022)	Custom (Olive Leaves)	MobileNet	98.43%	4.3	No

Based on the literature review, it is possible to conclude that the majority of studies used the PlantVillage dataset and also acquired real-time data equivalent to these plants. Furthermore, the authors (Ferentinos, 2018) classified plant diseases using 58 different plant species. According to the findings of these investigations, no research on the classification of diseases affecting mulberry leaves has been undertaken. The bulk of research employed TL models with a significant number of parameters, such as VGG16 with 138.4 million (M) parameters, and ResNet50 with 25.6M parameters, Xception with 22.9M parameters, DenseNet121 with 8.1M parameters, and so on, which need extensive GPU training time. Furthermore, several researchers spent a long time creating unique CNN models, such as 6.2 hours (Zhang et al., 2019) and 1 hour 56 minutes and 38 seconds (Akbar et al., 2022). Indeed, implementing these algorithms in low-power embedded devices, such as low-configuration Android mobiles, is particularly difficult. As a result, a lightweight model with fewer parameters and layers that require less training time than TL models are required to run on low-configuration Android mobiles. Additionally, no studies proved the use of explainable AI, such as SHAP or LIME, to focus on disease location in leaf images to explain the model’s interpretability.

## 3 Methodology

### 3.1 Proposed framework

**Figure 2:**
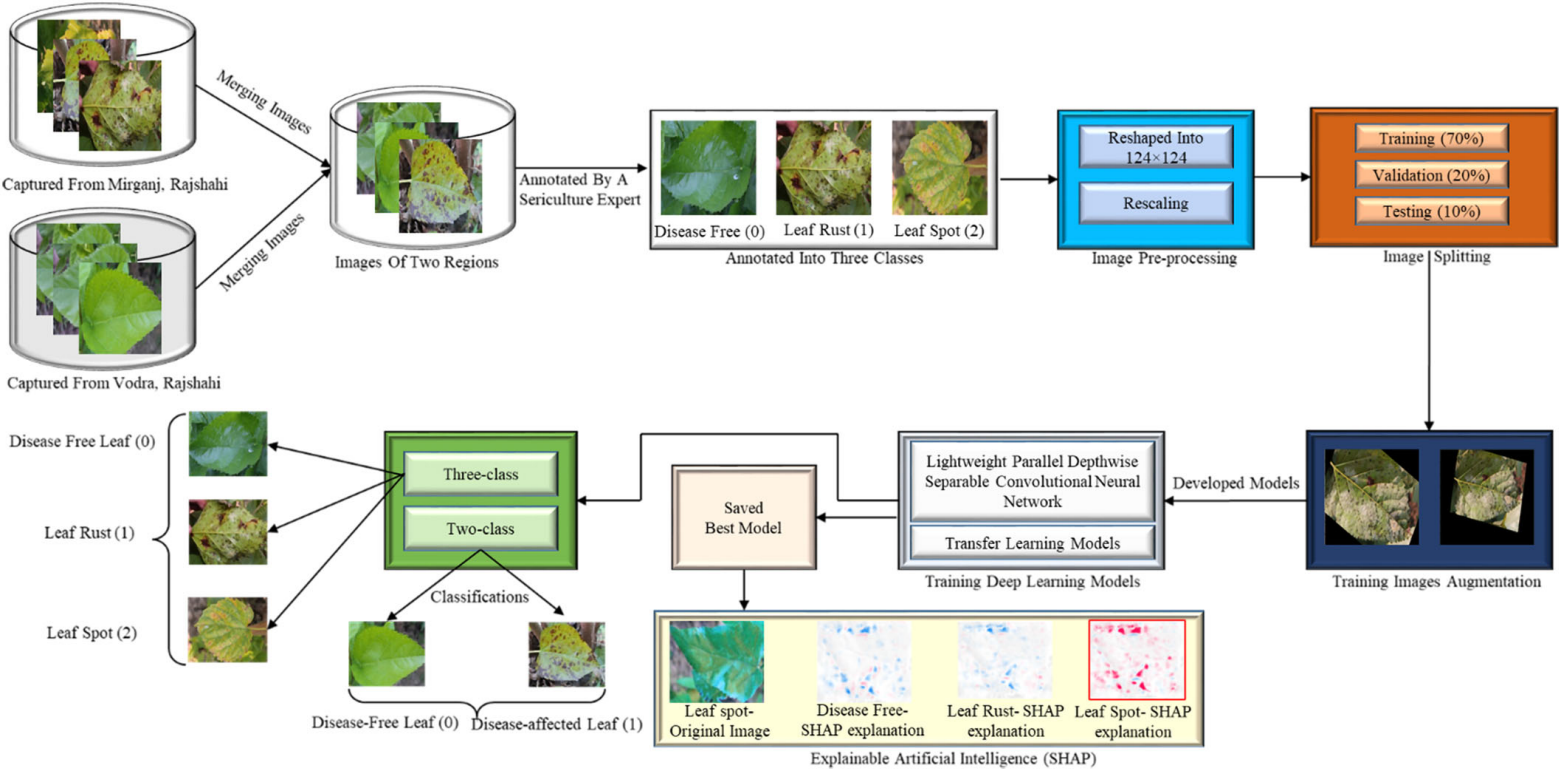
Proposed framework for mulberry leaf disease classification.

In response to the difficulties posed by mulberry leaf disease identification, a classification approach based on deep learning has been implemented. **Figure 2** depicts the three key processes of the proposed framework: generating an image dataset, applying deep learning for classification, and providing an interpretable model with SHAP. Since there is no dataset available for this domain, leaf images from two regions in Bangladesh with two leaf diseases were collected and labelled by a seasoned sericulture researcher. Following the collection and analysis of the images, the labelled data were separated into three categories (disease-free leaves, leaf rust, and leaf spot). In this work, a disease-affected leaf class was generated for the binary classification by assigning leaf rust and leaf spot to the same class. 70% of the images are used for training, 10% for validation, and 20% for testing the DL models using five-fold cross-validation. The images are then reshaped and pre-processed using standard techniques for normalization and enhancement. Then, a novel lightweight parallel depth-wise separable convolutional neural network (PDS-CNN) was created to categorize leaf diseases and SHAP was used to interpret disease location.

### 3.2 Mulberry leaf image acquisition

**Figure 3:**
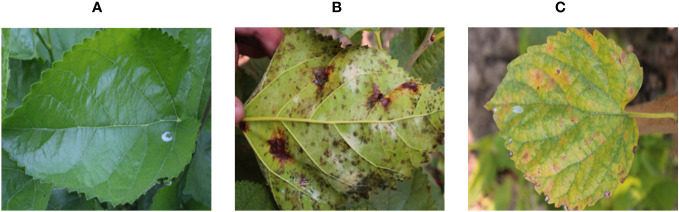
Samples of **(A)** disease-free leaf; **(B)** leaf rust; and **(C)** leaf spot.

Images of mulberry leaves are typically obtained using a digital camera or smartphone camera. The images may have been captured in a greenhouse, laboratory, or natural habitat. After consulting with researchers from the Bangladesh Sericulture Development Board (BSDB) in Rajshahi, Bangladesh, two certified and widespread mulberry leaf diseases (leaf spot and leaf rust) were selected for this experiment. In this study, images were acquired from mulberry gardens in Mirganj, Bagha, Rajshahi, and Vodra, Rajshahi, using a high-resolution DSLR camera in real-world situations. The mulberry dataset consists of a total of 1,091 images that have been classified by a sericulture expert into three classes: 440 healthy leaves, 489 leaves with leaf rust, and 162 leaves with leaf spots. Each leaf image has a resolution of 4,000 by 6,000 pixels. The sericulture experts were chosen from the main center of sericulture in Bangladesh with more than 10 years of experience in this field. **Figure 3** depicts a few examples of the acquired images.

### 3.3 Image pre-processing

The accuracy of the classification is directly influenced by the quality of the image preprocessing. This study simplifies the image processing stages so they can be easily implemented on embedded systems. During preprocessing, the images in the dataset are reduced to 124 pixels in width and height to reduce the need for more storage space and processing resources. Often, a high number of intensity values are used to depict an image. To simplify the complexity of the images, normalization is conducted, and the scale is modified from 0-255 to 0-1 by dividing the pixel values by 255.

### 3.4 Image augmentation

**Figure 4:**
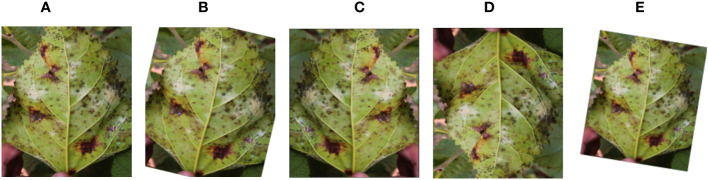
Samples of an **(A)** original image; **(B)** random rotation; **(C)** random horizontal flip; **(D)** random vertical flip; and **(E)** random affine.

The dataset is unbalanced, as shown in **Table 2**, where 764, 218 and 109 images were used for training, testing and validation respectively. As a result, different image augmentation techniques were used on the training images (**Figure 4A**) to balance the dataset. A random rotation of 30° was performed (**Figure 4B**). The images were randomly flipped by 50% in the horizontal and vertical orientations, as seen in **Figures 4C, D** In addition, as shown in **Figure 4E**, a random affine (degrees 5-15, translate 0.1-0.2, scale 0.7-0.8) was used. A total of 6,000 synthetic images were generated from the original 764 training images. Following image augmentation, a total of 6,000 and 4,000 training images for the three-class and two-class schemes, respectively, with 2,000 images for each class, were developed.

**Table 2:** The division of a dataset into training, testing, and validation sets for both multiclass and binary classes.

Schemes	Types	Training Set		Validation Set	Testing Set
		Before Augmentation	After Augmentation		
Scheme 1: Three-class	Disease-free leaf	308	2,000	44	88
	Leaf Rust	342	2,000	49	98
	Leaf Spot	114	2,000	16	32
	Total	764	6,000	109	218
Scheme 2: Binary	Disease-free leaf	308	2,000	44	88
	Disease-affected leaf	456	2,000	65	130
	Total	764	4,000	109	218

### 3.5 Customization of deep learning models

According to a review of the literature on the limits of various transfer learning (TL) models, the bulk of TL models have extremely large parameters, layers, and sizes, resulting in much longer computing power (Ferentinos, 2018; Chohan et al., 2020; Sanga et al., 2020; Chowdhury et al., 2021; Paymode and Malode, 2022). As a result, to address these difficulties, a simple, lightweight parallel depth-wise separable convolutional neural network with fewer parameters, layers, and size while requiring low overhead was designed in a customized form. A detailed explanation of the PDS-CNN model, as well as brief explanations of the state-of-the-art (SOTA) TL models employed in this study, are provided in the following subsections.

#### 3.5.1 Parallel depthwise separable convolutional neural network

**Figure 5:**
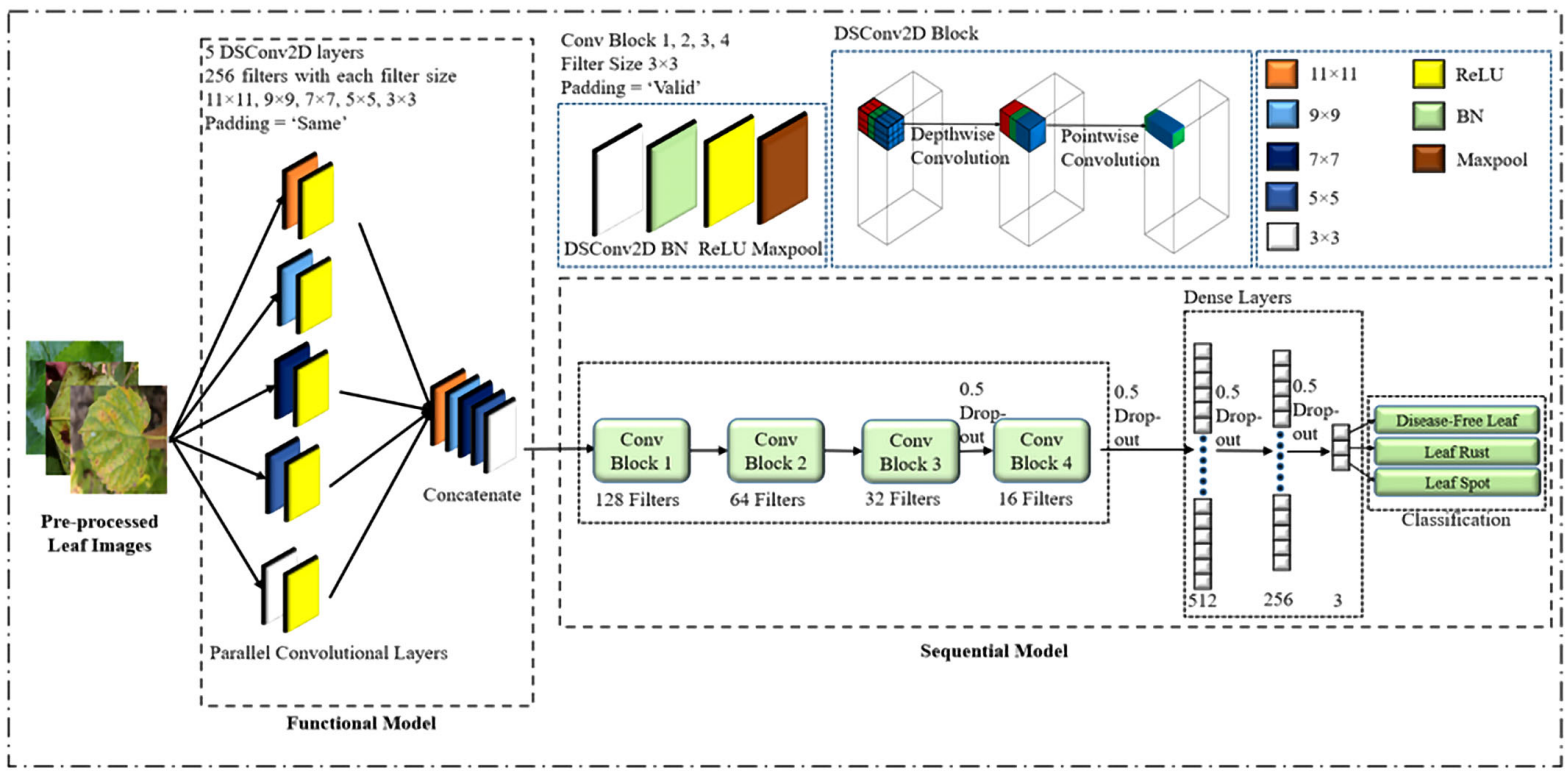
Proposed lightweight parallel depthwise separable convolutional neural network. (*DSConv2D means depthwise separable convolution and BN means batch normalization).

The specific objective was to create a CNN model that could efficiently extract the most significant characteristics with a small number of parameters and layers, allowing it to be used in a variety of real-world applications. However, if there aren’t enough parameters and layers, the model may fail to capture distinguishing features, and if there are too many, the model may overfit, resulting in a longer processing time. Taking these considerations into mind, a lightweight PDS-CNN model was created to extract discriminant features with low resources (small parameters, layers, and size). **Figure 5** depicts the lightweight PDS-CNN architecture for mulberry leaf disease classification.

Because a lightweight CNN model is proposed, the model is simplified in comparison to the TL models. The suggested model had nine convolutional layers (CL) and three fully connected layers (FC). The model would not have been able to extract the most critical features if only one CL had been utilized instead of five. In contrast, if five CLs are used sequentially, the number of layers (depth) increases, making the model more complex. As a result, the first five CLs were run in parallel, and their selection was dependent on trial and error. Each CL utilized a total of 256 kernels, with the first, second, third, fourth, and fifth kernel sizes being 11×11, 9×9, 7×7, 5×5, and 3×3, respectively. For picking the kernel size, our work followed the design of Krizhevsky et al., who employed big kernel sizes (such as 11×11, etc.) while ensuring appropriate classification performance (Krizhevsky et al., 2017; Nahiduzzaman et al., 2023a). Because different kernels produce distinct feature maps, different kernels were examined and combined, even if their sizes ranged from tiny to huge, to find notable features and achieve acceptable classification performance. The padding size was kept constant for the first five CLs to extract the relevant information in the border element of the mulberry leaf images. The feature maps generated by the concurrent CLs were then merged and fed into a sequential CL.

Furthermore, depthwise separable convolution (DSC) was employed instead of conventional convolution by dividing the typical convolution operation into two parts: a depthwise convolution and a pointwise convolution. First and foremost, a depthwise convolution applied a small kernel to a small section of an input feature map, resulting in a new feature map with the same number of channels. The depthwise convolution output is then passed through a pointwise convolution, where a 1×1 convolutional kernel is applied to each channel to create a new feature map with fewer channels. This DSC lowered the suggested CNN’s parameters from 2.2 million to 0.53 million (reducing computing complexity) and enhanced the classification performance of the proposed framework. Following the last four CLs, a batch normalization (BN) and a max-pooling layer with a 2×2 kernel were added to the processing chain. The sizes of the four CLs were set to 128, 64, 32, and 16, respectively, with 3×3 kernels, and the padding sizes were set to VALID. BN was used because it re-centers and re-scales each layer’s inputs, which speeds up and stabilizes the model’s execution. The ReLU activation function was used for all CLs. Aside from three fully connected (FC) layers, dropout was utilized to prevent overfitting and speed up the training process by disregarding 50% of all nodes at random. As a result, the proposed model has a sophisticated architecture comprising nine convolutional layers. The initial five layers operate in parallel, effectively functioning as a single layer in the overall structure. Following this unique configuration, four additional convolutional layers are incorporated, bringing the cumulative count to five convolutional layers. The model also includes three fully connected layers in conjunction with these convolutional layers. This intricate arrangement results in a total of eight distinct layers. In this investigation, two dropouts were used after the final two CLs and two more after the first two FC layers. In the final FC layer, the SoftMax activation function was employed to classify mulberry leaf disorders. There were three and two nodes in the final FC layer for multiclass and binary classifications, respectively. The loss function for the model was the sparse categorical cross-entropy loss function, and an ADAM optimizer with a learning rate of 0.001 was utilized. Ultimately, with a batch size of 32, the suggested lightweight PDS-CNN model was trained for 100 epochs. The PDS-CNN model is summarized in **Table 3**.

**Table 3:** An overview of the PDS-CNN model to classify mulberry leaf diseases.

Layer (Type)	Output Shape	Parameters
model (Functional)	(None, 124, 124, 1280)	5,975
DS_Conv6 (DS_Conv2D)	(None, 122, 122, 128)	175,488
bn1 (BatchNormalization)	(None, 122, 122, 128)	512
Av (Activation)	(None, 122, 122, 128)	0
mp1 (MaxPooling2D)	(None, 61, 61, 128)	0
DS_Conv7 (Conv2D)	(None, 59, 59, 64)	9,408
bn2 (BatchNormalization)	(None, 59, 59, 64)	256
av2 (Activation)	(None, 59, 59, 64)	0
mp2 (MaxPooling2D)	(None, 29, 29, 64)	0
DS_Conv8 (DS_Conv2D)	(None, 27, 27, 32)	2,656
bn3 (BatchNormalization)	(None, 27, 27, 32)	128
av3 (Activation)	(None, 27, 27, 32)	0
mp2 (MaxPooling2D)	(None, 13, 13, 32)	0
dp1 (Dropout)	(None, 13, 13, 32)	0
DS_Conv9 (DS_Conv2D)	(None, 11, 11, 16)	816
bn4 (BatchNormalization)	(None, 11, 11, 16)	64
av4 (Activation)	(None, 11, 11, 16)	0
mp3 (MaxPooling2D)	(None, 5, 5, 16)	0
dp2 (Dropout)	(None, 5, 5, 16)	0
ft (Flatten)	(None, 400)	0
Dense1 (Dense)	(None, 512)	205,312
bn3 (BatchNormalization)	(None, 512)	2,048
dp3 (Dropout)	(None, 512)	0
Dense2 (Dense)	(None, 256)	131,328
bn4 (BatchNormalization)	(None, 256)	1,024
dp4 (Dropout)	(None, 256)	0
Output (Dense)	(None, 3)	771
Total Parameters	535,786	
Trainable Parameters	533,770	
Non-trainable Parameters	2,016	

#### 3.5.2 Deep transfer learning models

**Figure 6:**
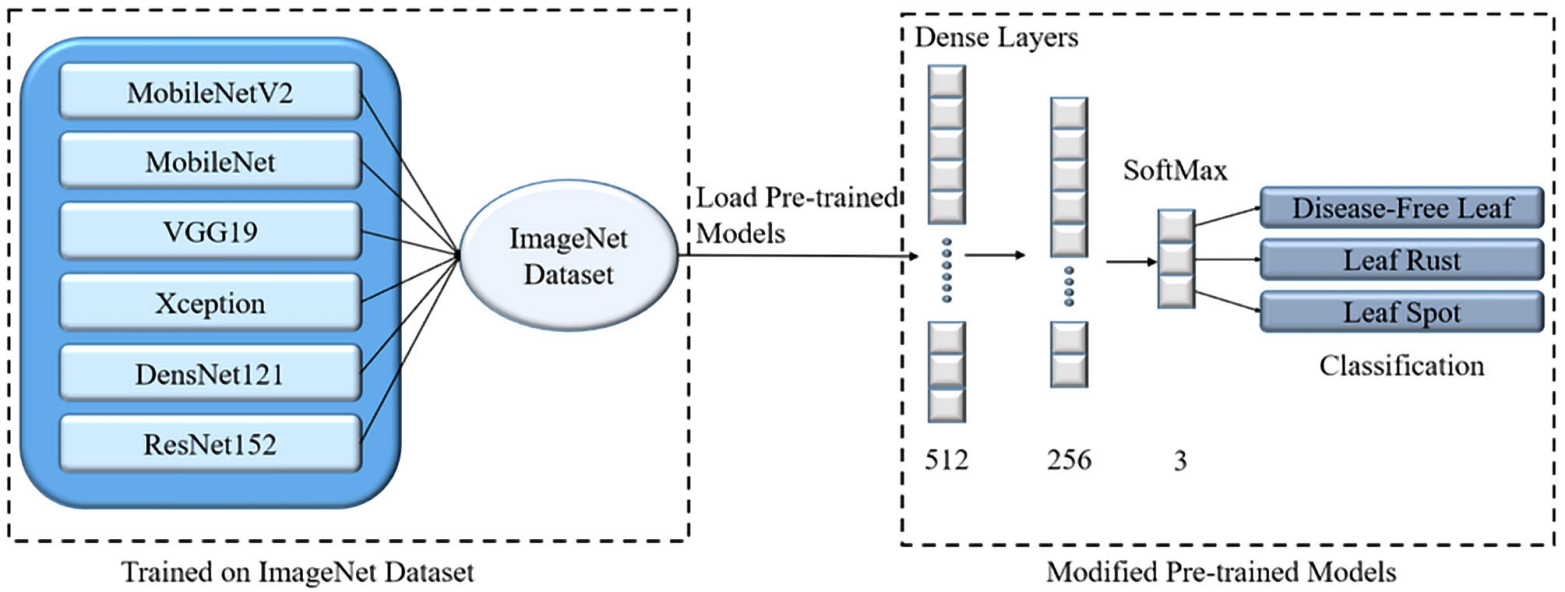
The modified architecture of transfer learning models to classify mulberry leaf diseases.

Transfer learning models have been effectively applied to a wide range of possible applications in recent years, including medical diagnosis and disease classification (Singh and Misra, 2017; Dhingra et al., 2019; Zhang et al., 2019; Chowdhury et al., 2020; Rahman et al., 2020a; Rahman et al., 2020b; Chowdhury et al., 2021; Nahiduzzaman et al., 2021b; Nahiduzzaman et al., 2021a; Qiblawey et al., 2021; Nahiduzzaman et al., 2023a; Nahiduzzaman et al., 2023b). Six TL models were used in this study: MobileNet (Howard et al., 2017), MobileNetV2 (Sandler et al., 2018), VGG19 (Simonyan and Zisserman, 2014), Xception (Chollet, 2017), DenseNet121 (Huang et al., 2017), and ResNet152 (He et al., 2016). All of these pre-trained models were trained using the ImageNet dataset, which comprises over 14 million images and 1,000 classifications. Following the loading of these models, the final layers were updated by adding three FC layers with 512, 256, and 3 nodes for identifying mulberry leaf disorders. **Figure 6** depicts the improved architecture of the TL models. These TL models were then trained for 100 epochs with a batch size of 32. In terms of classification outcomes and processing resources (parameters, layers, and sizes), the suggested novel lightweight PDS-CNN model was compared to the TL methods rather than earlier research (since no studies with the new mulberry dataset are available).

A concise explanation of these pre-trained networks is reported below. The Visual Geometry Group (VGG) at the University of Oxford introduced the VGG19 (Simonyan and Zisserman, 2014) CNN architecture in 2014. The architecture of VGG19 is made up of CLs with a huge number of filters per layer. After each convolutional layer comes to a max-pooling layer and a ReLU activation algorithm. Towards the end of the model, there are three fully connected layers, followed by a classification SoftMax activation function.

ResNet152 (He et al., 2016), a CNN architecture with deep depth and excellent accuracy, was developed by Microsoft Research in 2015. Due to the “vanishing gradients” problem, where the gradients of the parameters grow very small when the error is backpropagated through several layers, very deep neural networks have trouble learning. To address this issue, ResNet152 employs a residual connection to bypass one or more levels and connect a layer’s input to its output directly. Gradient flow is facilitated, allowing the network to learn effectively even at deep levels.

In 2016, Google released Xception (Chollet, 2017), which used depthwise separable convolutions. The entire input feature map is filtered by standard convolution to produce a single output feature map. A depthwise separable convolution applies the filter exclusively to the depth (channel) dimension of the input feature map, followed by a pointwise convolution to the output. This network can extract features from multiple channels separately, saving computation and memory.

In 2017, Google introduced the MobileNet (Howard et al., 2017) design, a CNN architecture that performs well on mobile and embedded devices with minimal processing capabilities. MobileNet employed a depthwise separable convolutional layer, which was generated by dividing a conventional convolution operation into depthwise and pointwise convolutions. By doing so, we may dramatically reduce the number of computations and parameters that the network must execute. MobileNetV2 (Sandler et al., 2018) is a MobileNet update. In actuality, MobileNet employs typical residual blocks with the same number of filters on the block’s input and output. In contrast, MobileNetV2 employs inverted residual blocks, which have different numbers of filters at the input and output of the block. As a result, MobileNetV2 has a reduced model size and can compute faster than MobileNet.

Huang et al. (2017) introduced DenseNet121, a densely connected convolutional network design, in 2017. It is a variant of the DenseNet structure, which employs a dense block structure with 121 layers, with the feature maps of all previous layers given into the current layer as inputs. As a result, information flows more smoothly across the network, and the issue of disappearing gradients is alleviated. DenseNet121 additionally employs 11 convolutional layers known as “bottleneck layers” to limit the number of feature mappings and control network expansion.

### 3.6 Explainable artificial intelligence

XAI in deep learning refers to the ability to comprehend and describe how a deep neural network operates and makes decisions (Lundberg, 2017). This is especially crucial for deep learning models, which can be ambiguous and challenging to understand. SHAP was utilized for the first time in this domain in this study to remove the “black box” nature of DL models, allowing the results from the PDS-CNN model to be further evaluated and explained so that sericulture professionals could use it in real-world scenarios. As a result, the model boosts their confidence when categorizing disease-free, leaf rust, and leaf spot leaves.

SHAP ranks the importance of model features by calculating the average of each feature value’s marginal contributions. The scores assigned to each pixel in a predicted image show the function of that pixel and can be used to clarify a categorization. The Shapley value was calculated using all conceivable combinations of mulberry leaf disease features. The Shapley values are pixelated, and the findings show that red pixels improve the likelihood of correctly identifying a class, whereas blue pixels lower it (Lundberg, 2017; Bhandari et al., 2022). The Shapley value was calculated using Equation (1).


(1) $$\emptyset_{\text{k}}=\;\sum_{\text{M}\subseteq \text{N}\backslash \text{k}}^{\text{k}}\frac{\text{M}!\left(\text{A}-\left|\text{M}\right|-1\right)!}{\text{A}!}\left[f_{x}\left(\text{M}\cup^{\;}\text{k}\right)-f_{x}\left(\text{M}\right)\right]$$

Where $f_{x}$ denotes the variation in output inclusion caused by Shapely values for a specific feature k . M is a subset of all features from feature N , excluding feature k . $\frac{M|\left(A-\left|M\right|-1\right)|}{A!}$ is the weighting factor that counts the number of permutations of the subset M . The predicted result, denoted by $f_{x}\left(M\right)$ , is derived from equation (2).


(2) $$f_{x}\;\left(\text{M}\right)=\text{P}[\text{f}\left(\text{x})\right|x_{M}]$$

SHAP replaces each original characteristic ( $x_{k}$ ) with a binary variable ( $b_{k}^{'}$ ) that indicates whether $x_{k}$ is present or absent, as demonstrated in Eq. (3)


(3) $$\text{l}\;\left({\text{b}}^{\prime }\right)=\emptyset_{0}+\;\sum_{\text{k}=1}^{\text{A}}\emptyset_{\text{k}}b_{k}^{\prime }$$

Where $\emptyset_{0}$ denotes the bias, $\emptyset_{k}b_{k}^{'}$ represents the contribution of the feature, A represents the number of simplified input features, and $l\left(b^{\prime }\right)$ is the substitute model for the proposed framework f(x) . The extent to which the presence of feature k contributes to the final result and $\emptyset_{k}$ assists in comprehension of the actual model.

## 4 Assessment metrics and implementation

The efficacy of the lightweight PDS-CNN model was estimated using a confusion matrix (CM). The following equations were used to calculate the accuracy, precision, recall, f1-score, and area under the curve (AUC) from the CM (Swets, 1988; Powers, 2020).


(4) $$\text{Accuracy}\;=\;\frac{T_{P}+T_{N}\;}{T_{P}+T_{N}+F_{P}+F_{N}}\;\;\;$$

(5) $$\text{Precision}\;=\;\frac{T_{P}}{T_{P}+F_{P}}$$

(6) $$\text{Recall}\;=\;\frac{T_{P}}{T_{P}+F_{N}}$$

(7) $$\text{F}1-\text{Score}\;=\;\frac{2\times \left(\text{Precision}\times \text{Recall}\right)}{\text{Precision}+\text{Recall}}$$

(8) $$\text{AUC}\;=\;\frac{1}{2}\left(\frac{T_{P}}{T_{P}+F_{N}}\;+\;\frac{T_{N}}{T_{N}+F_{P}}\right)$$

Where true positives, true negatives, false positives, and false negatives were represented by $T_{P}$ , $T_{N}$ , $F_{P}$ and $F_{N}$ , respectively. Keras was utilized to implement all deep learning algorithms and XAI, with TensorFlow as the backend running on the software PyCharm Community Edition (2021.2.3). A PC with 11th generation Intel(R) Core (TM) i9-11900 CPU @2.50GHz, 128GB RAM, and an NVIDIA GeForce RTX 3090 24 GB GPU running 64-bit Windows 10 Pro was used for model training and testing.

## 5 Results and discussion

In this study, both three-class and binary classifications were considered with a 5-fold CV and SHAP to assess the performance of the DL models.

### 5.1 Scheme 1: result for three-class classification

#### 5.1.1 Custom CNN models

**Figure 7:**
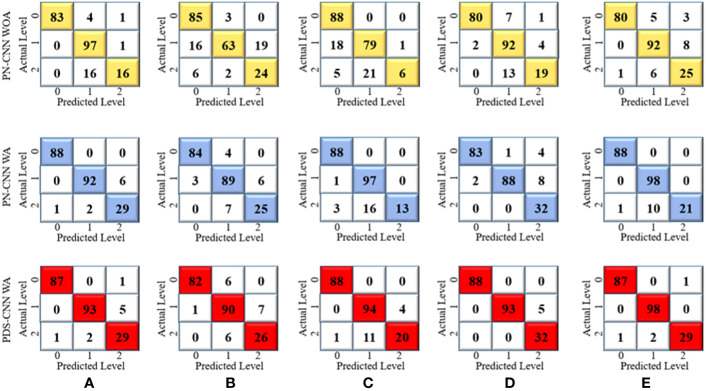
Confusion metrices for three-class classification of **(A)** Fold 1, **(B)** Fold 2, **(C)** Fold 3, **(D)** Fold 4, and **(E)** Fold 5.

First, a parallel CNN (PN-CNN) model without DSC was developed and trained using 764 images from disease-free leaves (0), leaf rust (1), and leaf spot (2), with 308, 342, and 114 images, respectively. This PN-CNN model without augmentation (PN-CNN WOA) was tested and validated using 218 images (88 disease-free leaves, 98 leaves with leaf rust, and 32 leaves with leaf spots) and 109 images (44 disease-free leaves, 49 leaves with leaf rust, and 16 leaves with leaf spots). The confusion matrix presented in **Figure 7** was used to conduct class-specific performance assessments. As indicated in **Table 4**, the average test accuracy, precision, and recall were 85.04 ± 4.89%, 84.0 ± 5.10%, and 78.8 ± 6.93% over the 5-fold mulberry dataset. Several conventional image augmentation approaches were used to improve the performance of the PN-CNN model. Each class had 2,000 images after augmentation, for a total of 6,000 images used to train the PN-CNN WA (with augmentation) model. The same images were utilized for validation and testing the model, and the results were promising, with average accuracy, precision, and recall of 93.12 ± 2.1%, 92.8 ± 2.93%, and 89.20 ± 5.34%, as shown in **Table 4**.

**Table 4:** Multiclass classification performance for comparison of PN-CNN WOA (without augmentation), PN-CNN, and PDS-CNN WA.

Model Name	Metrices	Fold 1	Fold 2	Fold 3	Fold 4	Fold 5	Average µ±σ(%)
PN-CNN WOA	Precision	0.91	0.76	0.81	0.86	0.86	84.0±5.10
	Recall	0.81	0.79	0.66	0.81	0.87	78.8±6.93
	F1-Score	0.84	0.76	0.66	0.83	0.86	79.0±7.32
	AUC (%)	95.75	91.64	91.71	97.98	97.11	94.84±2.68
	Accuracy (%)	89.91	78.89	79.36	87.61	89.45	85.04±4.89
PN-CNN WA	Precision	**0.94**	**0.89**	**0.94**	0.90	0.97	92.80±2.93
	Recall	0.94	0.88	0.80	0.95	0.89	89.20±5.34
	F1-Score	**0.94**	0.88	0.82	0.91	0.91	89.20±4.1
	AUC (%)	99.25	97.92	96.79	99.27	99.18	98.48±0.99
	Accuracy (%)	**95.87**	90.82	90.82	93.12	94.97	93.12±2.1
PDS-CNN WA	Precision	0.93	**0.89**	0.91	**0.95**	**0.98**	**93.20±3.12**
	Recall	**0.95**	**0.89**	**0.86**	**0.98**	**0.96**	**92.80±4.53**
	F1-Score	**0.94**	**0.89**	**0.88**	**0.97**	**0.97**	**93.00±3.85**
	AUC	**99.46**	**97.94**	**96.81**	**99.90**	**99.87**	**98.79±1.22**
	Accuracy	**95.87**	**90.83**	**92.66**	**97.71**	**98.17**	**95.05±2.86**

Bold results represent the best results for a particular metric among different models (with augmentation).

**Figure 8:**
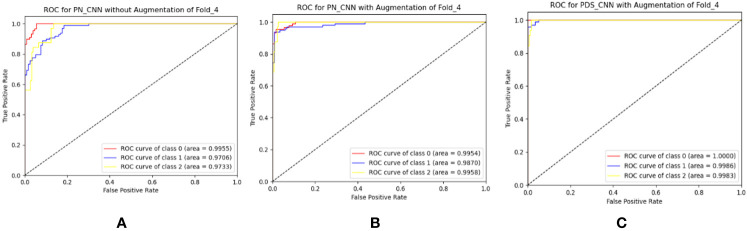
Best ROC for three-class classification of **(A)** PN-CNN without augmentation, **(B)** PN-CNN with augmentation, and **(C)** PDS-CNN with augmentation.

The suggested PN-CNN model comprises 2.2 million (M) parameters and is 24.5 megabytes (MB) in size. To minimize model parameters and size, the normal CLs were replaced by depth-wise separable CLs. This change lowered the resources (parameters and size) while improving classification performance. With parameters of only 0.53 M (nearly one-fourth of PN-CNN) and six times less (6.4 MB) than the PN-CNN model, the suggested lightweight PDS-CNN achieved an average test accuracy of 95.05 ± 2.86% (2% higher than PN-CNN) and recall of 92.80 ± 4.53% (3.6% higher than PN-CNN). PDS-CNN had an AUC of 98.79 ± 1.22%, which was about 0.5% higher than the PN-CNN (98.48 ± 0.99%) model. The PDS-CNN model achieved a 99.90% class-wise ROC across three classes, demonstrating its greater discriminant competence over the other two models (the ROCs of the PN-CNN WOA and PN-CNN WA were 99.29% and 99.27%, respectively, which are shown in **Figure 8**).

#### 5.1.2 Deep pre-trained models

**Figure 9:**
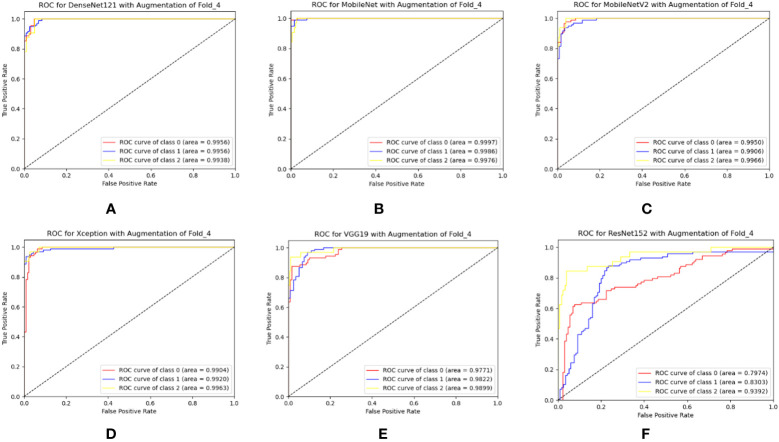
Best class-wise ROC for three-class classification of **(A)** DenseNet12, **(B)** MobileNet, **(C)** MobileNetV2, **(D)** Xception, **(E)** VGG19, and **(F)** ResNet152 with augmentation.

In this section, the six TL models were trained on 6,000 leaf images for three-class classification, with 2,000 images for each class. Actually, prior work has yet to be done on the mulberry leaf disease. As a result, the performance of the PDS-CNN model was compared to that of the six TL models to ensure that its performance (both in terms of classification performance and resource) is appropriate for this new dataset. All of the TL models were validated using the same images (109 leaf images), and the classification performance of the models was evaluated using 218 images. DenseNet121 has the highest average accuracy, precision, and recall scores of 93.12 ± 1.86%, 91.6 ± 2.65%, and 92.0 ± 3.16%, respectively. ResNet152, on the other hand, has the lowest accuracy, with accuracy, precision, and recall of 67.25 ± 5.00%, 64.6 ± 8.50%, and 62.8 ± 6.76%, respectively, as shown in **Table 5**. The average AUCs for DenseNet121, MobileNet, MobileNetV2, Xception, VGG19, and ResNet152 were 98.77 ± 0.88%, 97.72 ± 1.18%, 88.8 ± 2.99%, 95.42 ± 2.61%, 96.08 ± 1.92%, and 82.06 ± 4.25, respectively. As shown in **Figure 9**, all of the TL models achieved the best class-wise ROC for fold-4.

**Table 5:** Multiclass classification performance of six transfer learning models with augmentation.

Model Name	Metrices	Fold 1	Fold 2	Fold 3	Fold 4	Fold 5	Average µ±σ(%)
DenseNet121	Precision	**0.95**	0.87	0.91	0.92	**0.93**	**91.6±2.65**
	Recall	**0.95**	**0.92**	0.86	0.93	**0.94**	**92.00±3.16**
	F1-Score	**0.95**	**0.89**	0.88	0.93	**0.93**	**91.6±2.65**
	AUC (%)	**99.29**	**97.25**	**98.31**	99.49	**99.52**	**98.77±0.88**
	Accuracy (%)	**95.87**	90.83	91.28	94.04	**93.58**	**93.12±1.86**
MobileNet	Precision	0.90	**0.88**	**0.95**	**0.96**	0.88	91.4±3.44
	Recall	0.85	0.91	**0.87**	**0.96**	0.89	89.6±3.77
	F1-Score	0.87	0.89	**0.90**	**0.96**	0.89	90.2±3.06
	AUC (%)	97.03	96.83	96.77	**99.86**	98.13	97.72±1.18
	Accuracy (%)	91.74	**92.20**	**92.66**	**96.79**	91.74	93.03±1.91
MobileNetV2	Precision	0.91	0.84	0.90	0.93	0.92	90.00±3.16
	Recall	0.88	0.88	0.84	0.92	0.90	88.4±2.65
	F1-Score	0.89	0.85	0.86	0.93	0.91	88.8±2.99
	AUC (%)	97.79	96.07	95.84	99.41	99.51	97.72±1.57
	Accuracy (%)	93.12	88.53	88.99	93.12	93.12	91.38±2.14
Xception	Precision	0.83	0.80	0.89	0.91	0.90	86.6±4.32
	Recall	0.80	0.82	0.85	0.94	0.90	86.2±5.15
	F1-Score	0.81	0.81	0.86	0.92	0.90	86.00±4.52
	AUC (%)	94.46	93.27	92.47	99.29	97.61	95.42±2.61
	Accuracy (%)	86.69	85.32	90.37	93.12	92.20	89.54±3.05
VGG19	Precision	0.86	0.82	0.84	0.93	0.88	86.6±3.77
	Recall	0.82	0.83	0.80	0.92	0.86	84.6±4.18
	F1-Score	0.84	0.82	0.82	0.92	0.86	85.2±3.71
	AUC (%)	95.58	95.00	93.33	98.31	98.18	96.08±1.92
	Accuracy (%)	89.45	84.40	83.94	92.20	89.45	87.89±3.20
ResNet152	Precision	0.49	0.66	0.66	0.67	0.75	64.6±8.50
	Recall	0.53	0.65	0.57	0.68	0.71	62.8±6.76
	F1-Score	0.50	0.65	0.58	0.64	0.72	61.8±7.39
	AUC (%)	77.97	85.44	75.87	85.56	85.45	82.06±4.25
	Accuracy (%)	67.88	69.27	61.47	62.39	75.23	67.25±5.00

Bold results represent the best results for a particular metric among different models.

### 5.2 Scheme 2: results for binary classification

#### 5.2.1 Custom CNN models

**Figure 10:**
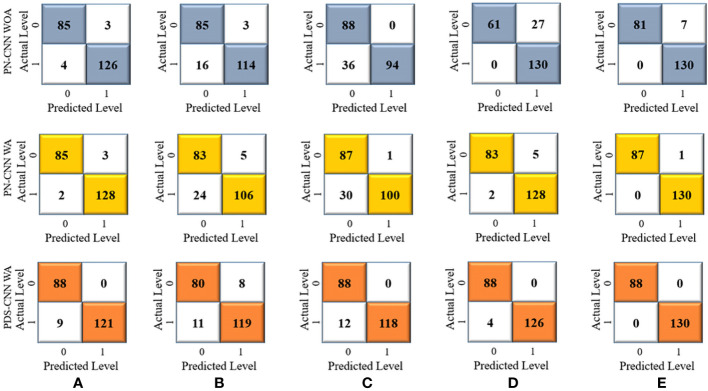
Confusion metrices for the binary classification of **(A)** Fold 1, **(B)** Fold 2, **(C)** Fold 3, **(D)** Fold 4, and **(E)** Fold 5.

**Figure 11:**
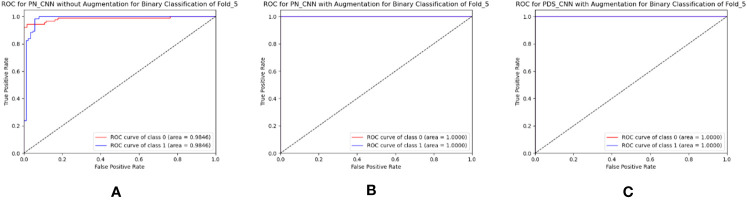
Best ROC for binary classification of **(A)** PN-CNN without augmentation, **(B)** PN-CNN with augmentation, and **(C)** PDS-CNN with augmentation.

The same approach for multiclass classification was used for binary classification. The PN-CNN WOA (no augmentation) model was trained using 764 leaf images, 308 of which were disease-free and 456 of which were disease-affected (1). The model was validated using 109 images (44 of disease-free leaves and 65 of diseased leaves), and the performance was evaluated using 218 images (disease-free leaf: 88 and disease-affected leaf: 130). **Figure 10** shows how the CM was used to investigate the models’ categorization performance. The PN-CNN WOA model’s accuracy, precision, and recall were 91.65 ± 4.95%, 92.4 ± 4.45%, and 91.2 ± 4.96%, respectively. Image augmentation was used to improve the model’s performance. Image augmentation was utilized to create 4,000 images after merging the two classes (leaf rust and leaf spot), with 2,000 images from each class used to train the model. The PN-CNN WA model enhanced testing accuracy by about 2% (93.30 ± 5.85%) when validated and tested with the same number of images. The PN-CNN WA (with augmentation) required more resources (more parameters, layers, and size) to distinguish between disease-affected and disease-free leaves. As a result, PDS-CNN was also used to do the binary classification. **Table 6** shows that the suggested lightweight PDS-CNN model achieved an optimistic accuracy of 96.06 ± 3.01% (nearly 3% higher than the PN-CNN WA model). Other than that, the recall rose by over 3% (96.2 ± 3.06%) over the PN-CNN WA model with lower resources. The average AUC for PN-CNN WA is slightly lower (98.71 ± 0.84%) than for PN-CNN WOA (98.32 ± 1.91%). Still, the PDS-CNN model achieved a promising AUC of 99.42 ± 1.04%, which is nearly 1% higher than the PN-CNN WOA model, demonstrating that it has a better discriminant capability than the other two models. Based on these findings, it is possible to conclude that the proposed PDS-CNN model is resilient for both binary and multiclass classifications. The fold-5 produced the best class-wise ROC, which was 100% for both models, as shown in **Figure 11**.

**Table 6:** Binary classification performance for comparison of PN-CNN WOA (without augmentation), PN-CNN, and PDS-CNN WA (with augmentation).

Model Name	Metrices	Fold 1	Fold 2	Fold 3	Fold 4	Fold 5	Average µ±σ(%)
PN-CNN WOA	Precision	0.97	**0.92**	0.85	0.91	0.97	92.4±4.45
	Recall	0.97	**0.92**	0.86	0.85	0.96	91.2±4.96
	F1-Score	0.97	**0.92**	0.86	0.85	0.96	91.2±4.96
	AUC (%)	99.14	97.26	98.92	99.76	98.46	98.71±0.84
	Accuracy (%)	96.79	**91.28**	83.49	89.91	96.79	91.65±4.95
PN-CNN WA	Precision	**0.98**	0.87	0.87	0.97	1.00	93.8±5.64
	Recall	**0.98**	0.88	0.88	0.96	0.99	93.8±4.83
	F1-Score	**0.98**	0.87	0.86	0.97	0.99	93.4±5.68
	AUC (%)	99.55	95	97.36	99.69	100	98.32±1.91
	Accuracy (%)	**97.71**	86.70	85.77	96.79	99.54	93.30±5.85
PDS-CNN WA	Precision	0.96	0.91	**0.94**	**0.98**	**1.00**	**95.8±3.12**
	Recall	0.97	0.91	**0.95**	**0.98**	**1.00**	**96.2±3.06**
	F1-Score	0.96	0.91	**0.94**	**0.98**	**1.00**	**95.8±3.12**
	AUC	**99.86**	**97.34**	**99.91**	**99.97**	**100**	**99.42±1.04**
	Accuracy	96.33	**91.28**	**94.50**	**98.17**	**100**	**96.06±3.01**

Bold results represent the best results for a particular metric among different models.

#### 5.2.2 Deep pre-trained models

**Figure 12:**
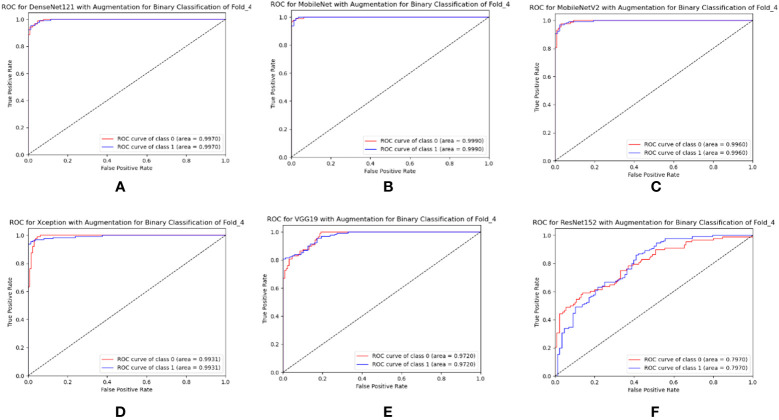
Best class-wise ROC for binary classification of **(A)** DenseNet12, **(B)** MobileNet, **(C)** MobileNetV2, **(D)** Xception, **(E)** VGG19, and **(F)** ResNet152 with augmentation.

The same 4,000 images of leaves were utilized to train the custom CNN model as well as the six TL models used for binary classification in this case. All of the TL models were validated using the same set of 109 leaf images, and their classification accuracy was evaluated across 218 images. DenseNet121 achieved the best overall accuracy (98.78 ± 1.04%), precision (94.2 ± 2.93%), and recall (94.8 ± 2.48%). ResNet152 has the lowest accuracy, precision, and recall, with values of 71.84 ± 5.48%, 73.8 ± 6.31%, and 7.00 ± 2.97%, respectively, according to **Table 7**. DenseNet121 had an AUC of 98.78 ± 1.04%, MobileNet had an AUC of 97.89 ± 1.88%, MobileNetV2 had an AUC of 98.25 ± 1.12%, Xception had an AUC of 95.13 ± 5.48%, VGG19 had an AUC of 95.99 ± 2.55%, and ResNet152 had an AUC of 79.32 ± 5.26%. All TL models had the best class-wise ROC for fold-4, as shown in **Figure 12**.

**Table 7:** Binary classification performance of six transfer learning models with augmentation.

Model Name	Metrices	Fold 1	Fold 2	Fold 3	Fold 4	Fold 5	Average µ±σ(%)
DenseNet121	Precision	**0.89**	**0.94**	0.97	0.94	**0.97**	**94.2±2.93**
	Recall	**0.90**	**0.95**	0.96	0.96	**0.97**	**94.8±2.48**
	F1-Score	**0.89**	**0.94**	0.97	0.95	**0.97**	**94.4±2.94**
	AUC (%)	**97.13**	97.99	**99.79**	99.70	**99.27**	**98.78±1.04**
	Accuracy (%)	**89.45**	**94.49**	96.79	94.95	**96.79**	**94.49±2.69**
MobileNet	Precision	0.86	0.92	**0.99**	**0.99**	0.93	93.8±4.87
	Recall	0.87	0.87	**0.98**	**0.98**	0.90	92.00±5.02
	F1-Score	0.85	0.89	**0.99**	**0.99**	0.91	92.6±5.57
	AUC (%)	94.79	**98.20**	99.63	**99.89**	96.96	97.89±1.88
	Accuracy (%)	85.32	89.45	**98.62**	**98.62**	91.74	92.75±5.22
MobileNetV2	Precision	0.88	**0.94**	0.90	0.97	0.96	93.00±3.46
	Recall	0.89	0.93	0.91	0.97	0.94	92.8±2.71
	F1-Score	0.88	0.93	0.90	0.97	0.95	92.6±3.26
	AUC (%)	96.82	97.20	98.20	99.60	99.41	98.25±1.12
	Accuracy (%)	88.07	93.58	89.91	96.79	94.95	92.66±3.22
Xception	Precision	0.73	0.87	0.92	0.97	0.95	88.8±8.59
	Recall	0.74	0.87	0.93	0.97	0.95	89.2±8.30
	F1-Score	0.73	0.87	0.92	0.97	0.95	88.8±8.59
	AUC (%)	84.41	95.83	97.88	99.31	98.22	95.13±5.48
	Accuracy (%)	72.94	87.16	92.66	96.79	94.95	88.9±8.61
VGG19	Precision	0.82	0.84	0.90	0.88	0.96	88.00±4.89
	Recall	0.84	0.82	0.90	0.88	0.95	87.8±4.58
	F1-Score	0.84	0.82	0.90	0.88	0.95	87.8±4.58
	AUC (%)	91.81	94.42	97.66	97.20	98.87	95.99±2.55
	Accuracy (%)	81.63	83.49	90.37	88.53	95.41	87.89±4.93
ResNet152	Precision	0.70	0.76	0.67	0.71	0.85	73.8±6.31
	Recall	0.68	0.74	0.68	0.70	0.75	71.00±2.97
	F1-Score	0.65	0.75	0.67	0.70	0.76	70.6±4.32
	AUC (%)	78.89	85.18	69.83	79.70	83.01	79.32±5.26
	Accuracy (%)	64.69	76.15	66.97	72.02	79.36	71.84±5.48

Bold results represent the best results for a particular metric among different models.

### 5.3 Performance comparison of PDS-CNN with SOTA TL models

**Figure 13:**
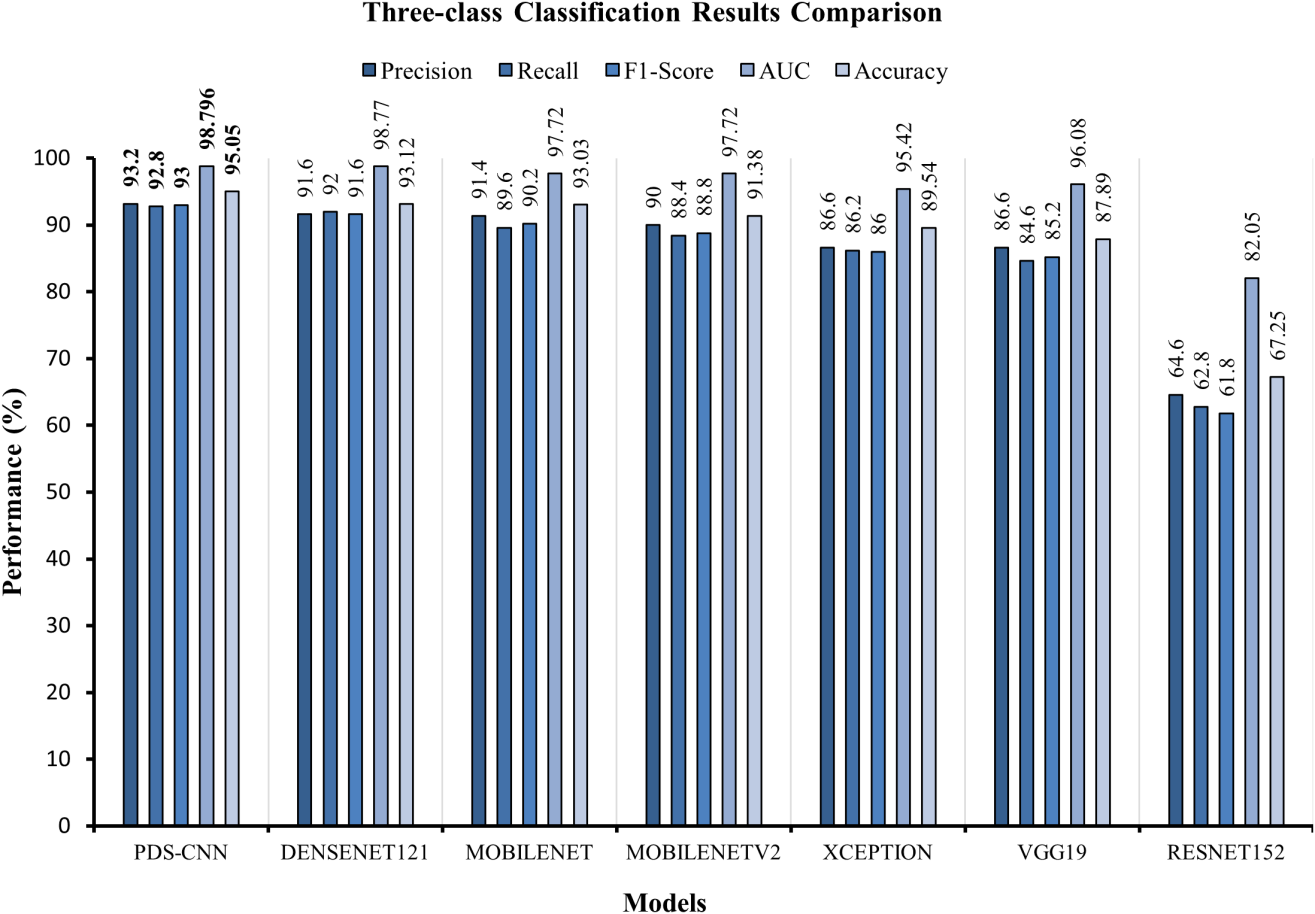
Graphical comparison of classification results for multiclass classification.

As indicated in **Table 8**, this section compares the performance of lightweight PDS-CNN with that of well-known TL models in terms of classification results and computational resources (parameters, layers, and sizes). DenseNet121, as previously stated, achieved the best classification results among the other five TL models for all three class and binary classifications. As shown in **Figure 13**, the suggested PDS-CNN achieved a reasonable accuracy of 95.05% for three-class classification, approximately 2% higher than the DenseNet121. Except for that, the AUC was 98.79%, which was higher than DenseNet121 (98.77%).

**Table 8:** Performance comparison for multi-class and binary classifications in terms of classification results and computational resources. For performance results, average values are mentioned with standard deviations.

Performance Criteria		PDS-CNN	DenseNet121	MobileNet	MobileNetV2	Xception	VGG19	ResNet152
Total Parameters (Million)		**0.535**	11.88	8	12.87	37.77	22.5	75.82
Trainable Parameters (Million)		**0.533**	4.8	4.8	10.61	16.9	2.49	16.9
Number of Layers		**8**	431	91	158	136	26	519
Size (Megabytes)		**6.3**	83.4	68	130	273	105	417
Three-class	Avg. Precision	**93.20±3.12**	91.6±2.65	91.4±3.44	90.00±3.16	86.6±4.32	86.6±3.77	64.6±8.50
	Avg. Recall	**92.80±4.53**	92.00±3.16	89.6±3.77	88.4±2.65	86.2±5.15	84.6±4.18	62.8±6.76
	Avg. F1-score	**93.00±3.85**	91.6±2.65	90.2±3.06	88.8±2.99	86.00±4.52	85.2±3.71	61.8±7.39
	Avg. AUC	**98.79±1.22**	98.77±0.88	97.72±1.18	97.72±1.57	95.42±2.61	96.08±1.92	82.06±4.25
	Avg. Accuracy	**95.05±2.86**	93.12±1.86	93.03±1.91	91.38±2.14	89.54±3.05	87.89±3.20	67.25±5.00
Binary	Avg. Precision	**95.8±3.12**	94.2±2.93	93.8±4.87	93.00±3.46	88.8±8.59	88.00±4.89	73.8±6.31
	Avg. Recall	**96.2±3.06**	94.8±2.48	92.00±5.02	92.8±2.71	89.2±8.30	87.8±4.58	71.00±2.97
	Avg. F1-score	**95.8±3.12**	94.4±2.94	92.6±5.57	92.6±3.26	88.8±8.59	87.8±4.58	70.6±4.32
	Avg. AUC	**99.42±1.04**	98.78±1.04	97.89±1.88	98.25±1.12	95.13±5.48	95.99±2.55	79.32±5.26
	Avg. Accuracy	**96.06±3.01**	94.2±2.93	92.75±5.22	92.66±3.22	88.9±8.61	87.89±4.93	71.84±5.48

Bold results represent the best results for a particular metric among different models.

**Figure 14:**
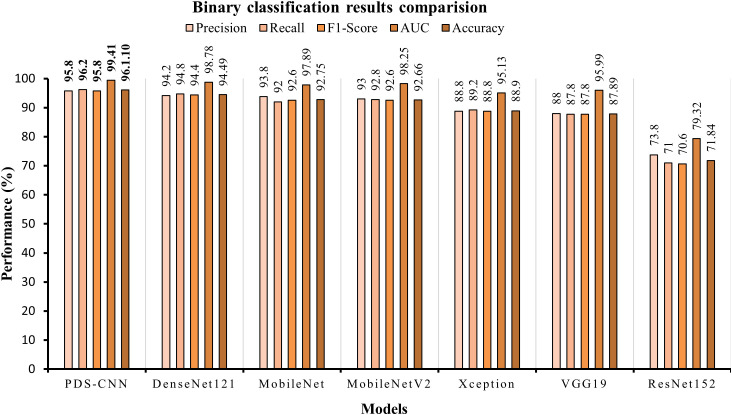
Graphical comparison of classification results for binary classification.

For binary classification, the PDS-CNN outperformed the DenseNet121 with accuracy, precision, and recall of 96.06% (almost 2% higher), 95.8% (almost 1.5% higher), and 96.2% (at most 2% higher). Furthermore, as shown in **Figure 14**, the AUC of the proposed PDS-CNN was nearly 1.0% (99.42%) higher than that of DenseNet121 (98.78%). Based on these findings, the lightweight PDS-CNN produced demonstrated promising discriminant capability across all six TL models.

**Figure 15:**
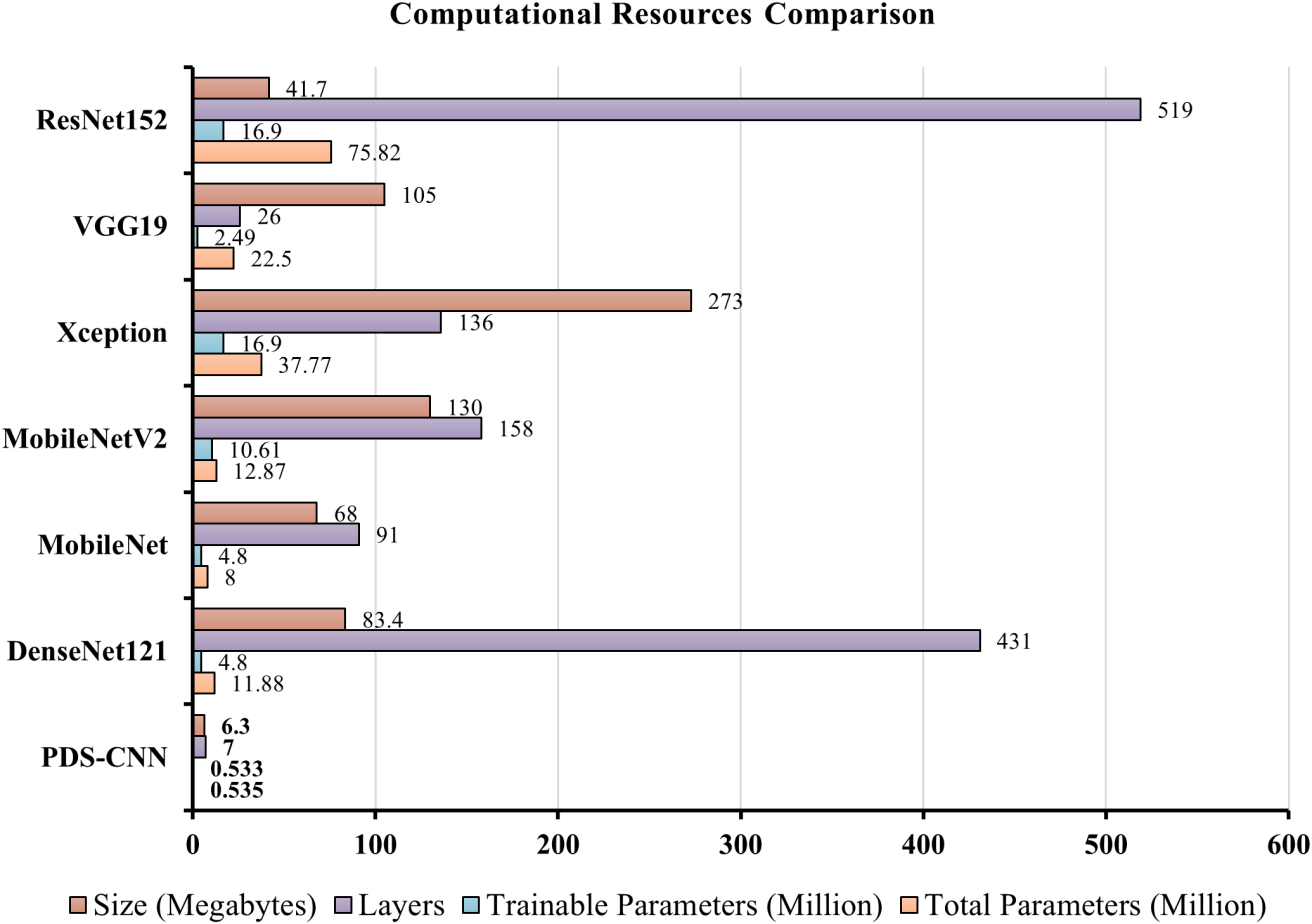
Computational resources comparison between the proposed PDS-CNN and TL models.

Additionally, with 91 layers and a size of 68 MB, MobileNet has the fewest total parameters (for this Mulberry dataset) of any TL model, but its accuracy (93.03% for three-class classification and 92.75% for binary classification) is not greater than DenesNet121. The proposed PDS-CNN model, on the other hand, has only 0.535 million, which is about 8 times smaller than MobileNet, and the proposed custom CNN model includes 8 layers (13 times less than MobileNet). Furthermore, the proposed lightweight model is only 6.3 MB in size, which is ten times lower than the MobileNet, as shown in **Figure 15**. Based on the findings, the suggested framework efficiently identified mulberry leaf diseases with higher accuracy, fewer factors, fewer layers, and lower overall size.

### 5.4 Interpretability PDS-CNN using SHAP

**Figure 16:**
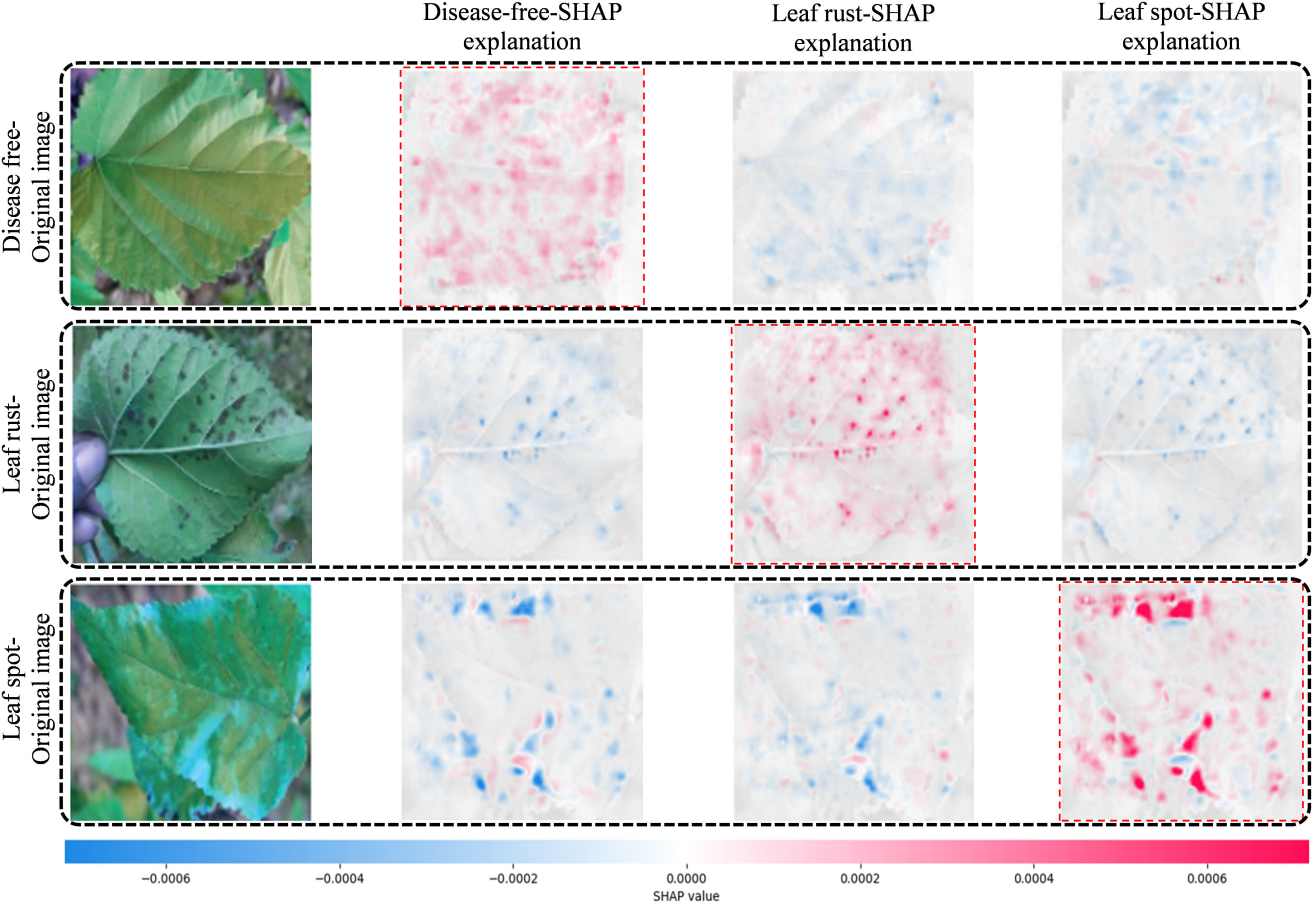
The sample images and the corresponding SHAP explanation images for the three classes.

The DL models are “black boxes” by definition. To address this issue and explain how the PDS-CNN model classifies the leaf disease by focusing on a specific region of the images, SHAP was incorporated into the proposed model for the first time in this work. The SHAP results (**Figure 16**) for a specific image offer explanation images for all three classes (disease-free leaf, leaf rust, and leaf spot). The relevant grey explanation backgrounds are almost invisible with the input images to the left. The first row reveals that the first explanation image has more red pixels, indicating that the leaf is disease-free. The lack of blue pixels in leaf rust and leaf spot SHAP explanation images, on the other hand, shows that the input image is not the leaf rust or the leaf spot. In the second row, however, the absence of red pixels in the SHAP explanation images of disease-free and leaf spots, as well as the presence of a large number of red pixels in the SHAP explanation image of leaf rust, accurately indicate that the image belongs to the leaf rust class. Similarly, in the third row, a high concentration of red pixels in the SHAP explanation image of a leaf spot and a high concentration of blue pixels in the SHAP explanation image of a disease-free leaf and leaf rust properly detect the original image containing the leaf spot disease. This visual explanation of the SHAP explanation images validates the model’s findings and gives the sericulture specialist or farmers concerned a clear indication of the mulberry diseases.

### 5.5 Discussion and future work

Although the architecture of the proposed lightweight PDS-CNN model is simple, it has just nine convolutional layers and three dense layers, with the first five CLs running in parallel (cutting nine CLs to five CLs) to ensure that the discriminant features were recorded. Yet, as demonstrated in **Table 8**, the model outperforms the other six models in classification performance. One of the key goals of this research was to create a model that improved classification results while reducing the number of parameters, layers, and size, which was accomplished by employing DS CLs instead of conventional CLs and making them suitable for use on embedded devices. Furthermore, SHAP has been introduced to ensure that the proposed model focuses on the correct disease-affected regions of an image rather than the other parts, making the model more readable to sericulture experts and assisting them in fast and accurate mulberry leaf disease classification, as well as assisting farmers in learning to distinguish one disease from another from these marked leaf images.

Due to a lack of leaf images, standard image augmentation methods were used to create synthetic images. As a result, this research will be expanded in the future by gathering more leaf images from all three classes and including a new class of powdery mildew leaf disease. As the model was hampered by an imbalanced data problem, a more reliable model will be built and applied on embedded systems such as mobile phones or raspberry pi to classify diseases directly from mulberry fields, which will benefit farmers and sericulture specialists.

The developed model can be applicable to identification of other leaf diseases and this will be considered for other major crops in Bangladesh.

## 6 Conclusion

An explanation generation (XAI) framework, in conjunction with a novel lightweight PDS-CNN model, is proposed in this paper for classifying disease-free leaf, leaf rust, and leaf spot from the newly made mulberry leaf images database. This XAI-based PDS-CNN model obtained 95.05 ± 2.86% accuracy for three-class classifications and 96.06 ± 3.01% accuracy for binary classifications with 0.53M parameters, 8 layers, and 6.3MB in size. The lightweight model achieved a promising classification performance while using fewer computational resources than the TL models, and the model’s interpretability was induced by SHAP and confirmed by sericulture experts, indicating that the proposed framework is capable of providing convincing and consistent results for mulberry leaf disease classification. Because of the model’s distinguishing features, it has the potential to be practiced by both sericulture professionals and farmers from rural areas. This has the potential to play a critical role in Bangladesh’s agriculture sector by assisting farmers in the early identification of mulberry leaf diseases, resulting in significant production savings and economic gain for farmers.

## Data availability statement

The original contributions presented in the study are included in the article/supplementary files, further inquiries can be directed to the corresponding author/s.

## Author contributions

MN: Conceived, designed and performed the analysis, written the paper. MC: Supervision, Conceived and Designed the analysis. AS and EN: Collected the Data. FA: Subject domain expert, collected the data and contributed analysis tool (annotation of the data). NA-E: Conceived and designed the analysis, interpretation of results, contributed in the analysis tool. MA: Literature review, Interpretation of results and discussion. AK: Conceived and designed the analysis, performed the analysis, contributed analysis tool (pipeline used in the investigation). JH: Wrote the initial draft and confirmed the methodology. All authors contributed to the article and approved the submitted version.
